# Clustering of integrin β cytoplasmic domains triggers nascent adhesion formation and reveals a protozoan origin of the integrin-talin interaction

**DOI:** 10.1038/s41598-019-42002-6

**Published:** 2019-04-05

**Authors:** Timo Baade, Christoph Paone, Adrian Baldrich, Christof R. Hauck

**Affiliations:** 1 Lehrstuhl Zellbiologie, Universitat Konstanz, 78457 Konstanz, Germany; 2 Konstanz Research School Chemical Biology, Universitat Konstanz, 78457 Konstanz, Germany

## Abstract

Integrins and integrin-dependent cell-matrix adhesions are essential for a number of physiological processes. Integrin function is tightly regulated via binding of cytoplasmic proteins to integrin intracellular domains. Yet, the complexity of cell-matrix adhesions in mammals, with more than 150 core adhesome proteins, complicates the analysis of integrin-associated protein complexes. Interestingly, the evolutionary origin of integrins dates back before the transition from unicellular life to complex multicellular animals. Though unicellular relatives of metazoa have a less complex adhesome, nothing is known about the initial steps of integrin activation and adhesion complex assembly in protozoa. Therefore, we developed a minimal, microscope-based system using chimeric integrins to investigate receptor-proximal events during focal adhesion assembly. Clustering of the human integrin β1 tail led to recruitment of talin, kindlin, and paxillin and mutation of the known talin binding site abolished recruitment of this protein. Proteins indirectly linked to integrins, such as vinculin, migfilin, p130^CAS^, or zyxin were not enriched around the integrin β1 tail. With the exception of integrin β4 and integrin β8, the cytoplasmic domains of all human integrin β subunits supported talin binding. Likewise, the cytoplasmic domains of integrin β subunits expressed by the protozoan *Capsaspora owczarzaki* readily recruited talin and this interaction was based on an evolutionary conserved NPXY/F amino acid motif. The results we present here validate the use of our novel microscopic assay to uncover details of integrin-based protein-protein interactions in a cellular context and suggest that talin binding to integrin β cytoplasmic tails is an ancient feature of integrin regulation.

## Introduction

The transition from unicellular organisms to multicellular animals (metazoans) coincides with the emergence of dedicated cell adhesion molecules such as cadherins and integrins^1^. Since their discovery in the mid-80s, it became clear that integrins are not only essential for coupling of the cytoskeleton to the extracellular matrix upon cell attachment, but that they also contribute to a multitude of basic cellular processes including cell survival, proliferation, differentiation, and cell migration. It is therefore not surprising that these heterodimeric transmembrane glycoproteins are critical in physiological and pathological settings such as tissue development and homeostasis, immune response, wound healing, and cancer metastasis^2,3^. Encoded in the human genome are 18 integrin α and 8 integrin β subunits forming 24 distinct heterodimers^2,4^. Upon ligand binding, integrins undergo a conformational change leading to the disruption of a salt bridge between the transmembrane domains of the two subunits and consecutive stabilization of the open, active conformation, where the cytoplasmic domains of the α and β subunits are spatially separated^5^. This process is termed outside-in activation and contrasts integrin inside-out activation, where cytoplasmic events, e.g. binding of integrin activators to the integrin β subunit cytoplasmic domain initiate the open, active conformation^6,7^. One of the proteins able to initiate integrin inside-out activation is talin. With its N-terminal FERM domain, talin binds to the NPXY/F motif in the membrane proximal part of the integrin β cytoplasmic tail, thereby pushing apart the two integrin subunits followed by the unfolding of their ectodomains^8,9^. Upon talin binding to the integrin β subunit, additional cytoplasmic proteins are recruited to integrin-initiated focal adhesion (FA) sites^10^. Recent proteomic studies in mammalian cells have provided a comprehensive view of the integrin adhesome and revealed, that the full list of focal adhesion constituents contains more than 150 proteins^11^. Owing to this complexity, the processes regulating the assembly and disassembly of these dynamic protein complexes, the hierarchy of their recruitment, their stoichiometry as well as their 3-dimensional arrangement is still incompletely understood. In particular, the initial molecular events following integrin activation have been hard to resolve as multiple integrin heterodimers with sometimes overlapping specificity for extracellular matrix proteins are present on the surface of any given cell type^2,12^. FA analysis has been further hampered by the spatial and temporal heterogeneity of these structures within a single cell and the current lack of an *in vitro* reconstituted system with recombinant purified components. These shortcomings prompted us to establish a minimal system with chimeric integrin β subunits, which can be clustered by multivalent ligands to stimulate initial protein recruitment. Moreover, we hypothesized that integrin-mediated outside-in signaling might be a conserved process allowing the investigation of the initial events in less complex, evolutionary ancient organisms.

Using the novel chimeric integrins we demonstrate here that ligand-induced clustering recapitulates the known recruitment of talin to a conserved site in the integrin β cytoplasmic tail. Furthermore, we observe differential recruitment of additional focal adhesion proteins including kindlin and paxillin but not vinculin, indicating direct versus indirect association of these cytoplasmic proteins with different integrin β subunits. Finally, this system allows us to test the evolutionary conservation of talin binding to integrin cytoplasmic domains encoded in the genome of unicellular protists such as *Capsaspora owczarzaki*. Our findings not only demonstrate the usefulness of chimeric integrins to resolve the initial molecular events at integrin cytoplasmic tails in a cellular context, but also provide first evidence that integrin activity regulation by talin evolved before the emergence of metazoans.

## Results

### Using bacteria-triggered integrin clustering to detect and quantify protein recruitment to the integrin cytoplasmic domain

**Figure 1:**
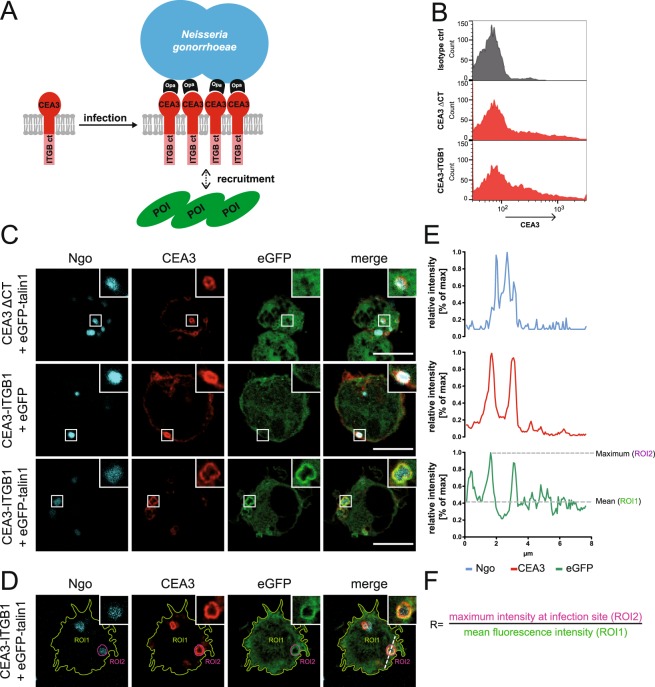
The OPTIC principle and quantification. (**A**) Schematic overview of the OPTIC workflow. Opa-expressing *N*. *gonorrhoeae* (Ngo) are used to cluster CEACAM3-integrin β cytoplasmic tail (ITGB ct) fusion proteins potentially resulting in the recruitment of an intracellular protein of interest (POI). (**B**) Transiently transfected 293 T cells were stained with a monoclonal α-CEACAM antibody and analysed by flow cytometry. As a control, cells were stained with an isotype-matched control antibody. (**C**) 293 T cells were transiently co-transfected with a CEACAM3-ITGB1 fusion construct (CEA3-ITGB1) or CEA3-ΔCT together with eGFP-talin1 or eGFP and seeded on poly-L-lysine. Cells were infected for 1 h with Pacific Blue-labeled *Neisseria gonorrhoeae*, fixed, and stained for CEACAM3. Bars represent 10 µm. (**D**) Representative micrograph showing the region of interests (ROI) selected for quantification. After encircling the perimeter of the cell (ROI1) the mean GFP fluorescence for ROI1 is determined. Next, the infection site (indicated by the outline of cell-attached bacteria) is defined as ROI2 and the maximum GFP fluorescence in ROI2 is determined. (**E**) Line scan (dashed line in **D**) shows the intensity distribution of Pacific Blue (Ngo; blue), CEACAM3 (Cy5, red) and GFP-talin (green). The horizontal lines indicate the mean GFP fluorescence intensity in ROI1 and the maximum GFP intensity in ROI2. (**F**) Formula used to calculate the ratio of recruitment R. A ratio of max. ROI2/mean ROI1 > 2 was defined as positive protein recruitment.

**Figure 2:**
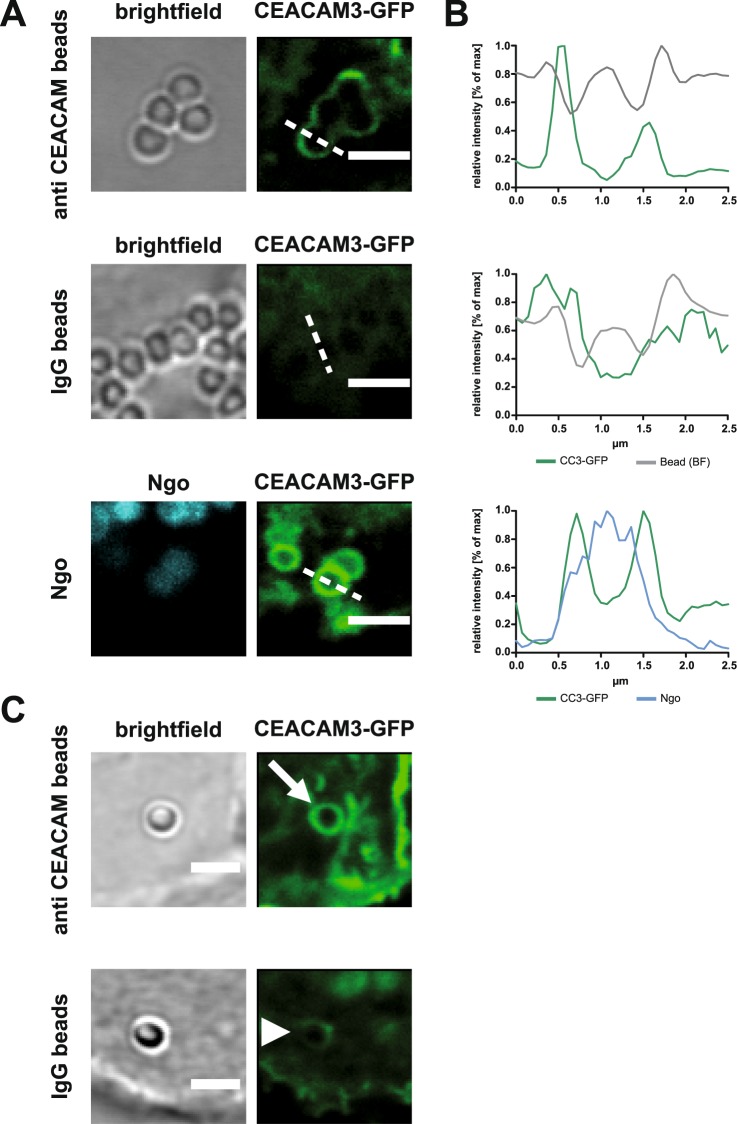
*Neisseria gonorrhoeae* can be substituted by α-CEACAM antibody conjugated polystyrene beads. (**A**) 293 T cells were transiently transfected with GFP-tagged full-length CEACAM3 and incubated for 1 h with polystyrene beads coated with a monoclonal α-CEACAM antibody or coated with an isotype-matched irrelevant monoclonal antibody (IgG beads). As a positive control, CEACAM3-expressing cells were infected for 1 h with Pacific Blue-labeled *N*. *gonorrhoeae* (Ngo). Images show representative micrographs of infection sites. (**B**) Line scans depict the GFP-fluorescence intensity along the dashed lines in a). Polystyrene beads coated with α-CEACAM antibody lead to comparable clustering of CEACAM3 as seen for Ngo, while IgG beads do not cluster the receptor. (**C**) Cells as in a) were incubated with beads sonicated prior to antibody coupling. Again, single beads coated with α-CEACAM antibody lead to clustering of CEACAM3 (white arrow), while IgG beads did not cluster the receptor (arrowhead).

Opa proteins of *Neisseria gonorrhoeae* (Ngo) bind with high affinity to the extracellular IgV-like domain of human CEACAM family members such as CEACAM3^13^. The multivalent bacteria induce local clustering of CEACAM3, which in turn rapidly recruits binding partners to the cytoplasmic domain of CEACAM3^14,15^. We reasoned that fusion of the intracellular part of the integrin β subunits with the extracellular IgV-like domain of CEACAM3 would allow the selective clustering of integrin β tails and the monitoring of recruitment of cytosolic integrin β binding partners. Therefore, a panel of chimeric CEACAM3-integrin fusion constructs were generated (Fig. 1A and Suppl. Fig. S1) and surface expression of the chimeric proteins was confirmed by flow cytometry (Fig. 1B). The integrin β1 cytoplasmic domain is known for its ability to recruit talin^16^. Therefore, talin recruitment to chimeric proteins was tested in transiently transfected 293T cells. Cells expressing CEACAM3-integrin β1 (CEA3-ITGB1) and GFP-talin were infected with Opa protein-expressing, CEACAM-binding Ngo for 1 h. As expected, Ngo attached to the cell surface and efficiently clustered the chimeric receptors (Fig. 1C). We therefore coined this method OPTIC for *O*pa *p*rotein *t*riggered *i*ntegrin *c*lustering. Importantly, bacteria-induced clustering of CEA3-ITGB1 also induced a strong enrichment of talin around the cell-associated bacteria (Fig. 1C). In contrast, a CEACAM3 protein lacking the cytoplasmic domain (CEA3-ΔCT) did not show any talin recruitment upon OPTIC (Fig. 1C), indicating that the observed talin recruitment is strictly dependent on the presence of the integrin cytoplasmic tail and is not mediated via other infection-associated events. There was no recruitment of GFP alone, demonstrating that the increased signal intensity seen for GFP-talin is not due to volume effects around the cell-associated bacteria (Fig. 1C). To quantify the extent of protein recruitment to clustered integrin cytoplasmic tails, two regions of interest (ROI) were defined: ROI1 circles the whole cell area, while ROI2 covers the clustered CEACAM3 at the infection site (Fig. 1D). The mean fluorescence intensity in ROI1 and the maximum fluorescence intensity in ROI2 are used to calculate the relative intensity ratio R (Fig. 1F). A value of R = 2 indicates a two-fold local enrichment over the mean cellular distribution, a value we use as threshold for positive recruitment. The enrichment of the protein of interest around cell-associated bacteria can also be nicely illustrated by plotting the fluorescence intensity values along a line indicated in Fig. 1D. Fluorescence intensity for each channel along the line is represented relative to maximum values and the overlapping maximum peaks of the red (CEA3-ITGB1) and green (GFP-talin1) signals highlight the recruitment of talin to clustered CEA3-ITGB1 (Fig. 1E). The dashed gray lines indicate the maximum (ROI2) and mean value (ROI1) of the ROIs marked in Fig. 1D. To make our approach not only quantitative, but also independent of pathogenic bacteria, we clustered CEA3-ITGB1 with latex beads coated with a monoclonal α-CEACAM antibody. Also in this case, we could observe clustering of the CEA3-ITGB1 chimera with α-CEACAM coated beads, while control beads coated with irrelevant IgG were not effective demonstrating that α-CEACAM coated beads could substitute for pathogenic bacteria in this assay (Fig. 2). Together, these results suggest that clustering of β-integrin chimeras can recapitulate the initial events occuring upon physiological stimulation of integrins.

### OPTIC captures the direct interaction of talin with the cytoplasmic tail of integrin β1

**Figure 3:**
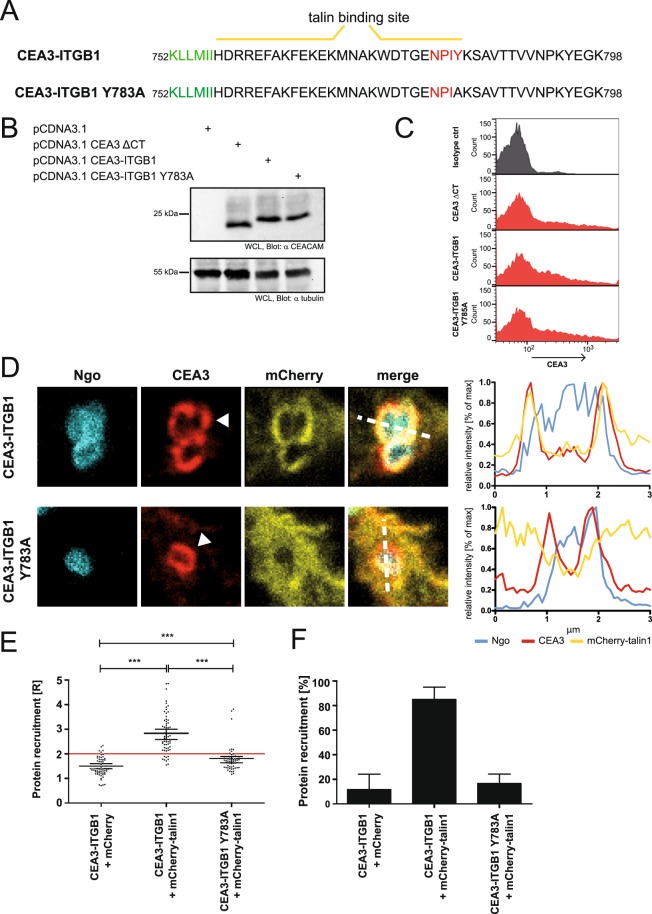
OPTIC can be used to visualize effects of point mutations on protein localization. (**A**) Amino acid sequence of the integrin β1 cytoplasmic tail indicating the talin binding sites (yellow lines). Residues marked in green are embedded in the plasma membrane. The main talin binding motif is highlighted in red. (**B**) 293 T cells were transiently transfected with the indicated expression constructs. Western Blot of whole cell lysates (WCL) with a monoclonal α-CEACAM antibody (upper panel) demonstrates equal amounts of CEA3-ΔCT, CEA3-ITGB, and CEA3-ITGB1 Y783A. Probing of the membrane with a monoclonal α-tubulin antibody confirms equal loading of the samples (lower panel). The full scans of both Western blots are shown in Suppl. Fig. S2. (**C**) Cells as in (**B**) were stained for CEACAM3 and analysed by flow cytometry. 15–20% of the transfected cell population expressed CEACAM3. (**D**) Cells were co-transfected with CEA3-ITGB1 or CEA3-ITGB1 Y783A together with mCherry-talin1. Transfected cells were infected for 1 h with Pacific Blue-labeled Ngo, fixed, and stained for CEACAM3. Bacterial binding to CEACAM3 resulted in clustering of CEA3-ITGB1 and CEA3-ITGB1 Y783A as evident in the CEACAM3 chanel (arrowheads). Line plots through infection sites (dashed lines in merged pictures) show the distribution of fluorescence intensities of CEACAM3, mCherry-talin1 around Pacific Blue-stained *Ngo*. Talin is not enriched upon clustering of CEA3-ITGB1 Y783A. (**E**) Quantification of talin recruitment in samples processed as in (**D**). Each data point reflects talin recruitment in a CEACAM3-expressing cell with CEACAM3-bound bacteria. Horizontal lines indicate mean values and 95% confidence intervals (whiskers) of n = 60 cells from three independent experiments. Statistically significant differences from the CEA3-ΔCT control were evaluated using one-way ANOVA, followed by Bonferroni post-hoc test (***p < 0.001). (**F**) Percentage of CEACAM3-expressing cells, which show a ratio of talin recruitment R ≥ 2. Bars indicate mean values ± standard deviation of n = 60 cells/sample.

As OPTIC was able to detect and quantify the local enrichment of talin around clustered chimeric receptors we wanted to investigate, if this recruitment depends on the known direct interaction with the cytoplasmic tail of integrin β1. The short amino acid sequence of the integrin β1 cytoplasmic tail comprises two known talin interaction sites, with the membrane proximal NPXY/F motif being the main binding site for talin^16^. Therefore, we introduced a point mutation in the integrin β1 cytoplasmic tail replacing tyrosine for alanine (Y783A) (Fig. 3A), a missense mutation known to impair talin association with integrin β1^17^. Similar expression levels of the CEA3-ITGB fusion proteins by the transfected cells were verified by Western blotting and flow cytometry (Fig. 3B,C). Upon infection with Opa-expressing Ngo, cells expressing the wildtype CEA3-ITGB1 construct showed the expected recruitment of talin1 (Fig. 3D) and the maximum intensity peaks of CEA3-ITGB1 and talin1 colocalized (Fig. 3D). Altering the NPXY motif tyrosine residue to an alanine lead to the loss of talin recruitment and a disruption of colocalization of the maximum peaks (Fig. 3D). To quantify the extent of talin recruitment the ratio R for 60 cells for each sample from three independent experiments was calculated. In the case of wildtype CEA3-ITGB1, a mean ratio of R = 2.84 was observed (Fig. 3E). Around 85% of the cells analysed were positive for talin recruitment (Fig. 3F). Talin recruitment by CEA3-ITGB1 was also significantly different to the negative control, which was transfected with mCherry (mean value of R = 1.5). Importantly, similar to the complete deletion of the cytoplasmic tail (Fig. 1C), the Y783A mutation severely impaired talin recruitment (Fig. 3F). In cells expressing CEA3-ITGB1 Y783A, a talin enrichment of R = 1.8 was observed, with only about 17% of the cells showing recruitment, which is comparable to the values seen for the mCherry expressing negative control (Fig. 3E,F). This experiment underscores that our microscopic approach is sensitive enough to detect the effect of single amino acid alterations on protein recruitment. Therefore, OPTIC can be used to verify biochemical data obtained with purified components on integrin-mediated protein-protein interactions in a complex cellular environment.

### Talin recruitment is a conserved feature of human integrin β CTs

**Figure 4:**
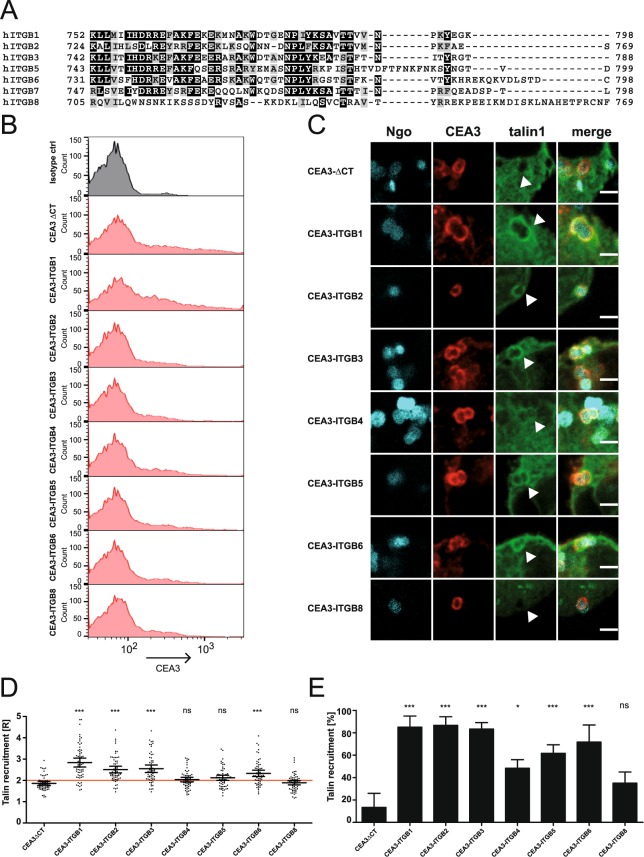
Talin is recruited to the cytoplasmic tail of most human integrin β subunits. (**A**) Amino acid sequence alignment of the human integrin β cytoplasmic tails including the highly conserved membrane proximal NPxY/F and membrane distal NxxY/F motifs. Identical residues are shaded in black; highly similar residues are shaded gray. (**B**) 293 T cells were transiently co-transfected with the indicated CEACAM3-integrin fusion constructs and GFP-talin1. Cells were stained for CEACAM3 and analysed by flow cytometry. (**C**) Cells as in (**B**) were infected for 1 h with Pacific Blue-labeled Ngo, fixed, and stained for CEACAM3. Micrographs show representative sites of bacterial attachment and CEA3-ITGB clustering. Bars represent 2 µm. (**D**) Talin recruitment to the different CEACAM3-integrin fusion proteins in (**C**) was evaluated. Each data point reflects the talin recruitment ratio R in a CEA3-ITGB-expressing cell with attached bacteria. Horizontal lines indicate mean values and 95% confidence intervals (whiskers) of n = 60 cells from three independent experiments. Statistically significant differences from the CEA3-ΔCT control were evaluated using one-way ANOVA, followed by Bonferroni post-hoc test (***p < 0.001; ns = not significant). (**E**) Percentage of CEA3-ITGB-expressing cells, which show a ratio of talin recruitment R ≥ 2. Statistical significance was calculated using one-way ANOVA, followed by Bonferroni post-hoc test (*p < 0.05, ***p < 0.001, ns = not significant).

As talin binding to integrin β1 has been well described before, we set out to compare talin recruitment to other human integrin β subunits in a cellular context. Sequence alignment revealed that, with the exception of integrin β4 and β8, all β-subunits share the highly conserved membrane proximal NPXY/F and the lesser conserved membrane distal NXXY motif (Fig. 4A). The cytoplasmic tail of integrin β4 was not included in the alignment due to its extraordinary length. CEACAM3-integrin β chimeras (β1–β8) were generated and, with the exception of the integrin β7 chimera, expressed on the cell surface as confirmed by flow cytometric analysis (Fig. 4B). Upon infection with CEACAM-binding bacteria, all CEA3-ITGB chimeras were clustered at equivalent levels by the CEACAM-binding bacteria (Fig. 4C and Supplementary Fig. S2). However, there were clear differences in talin recruitment (Fig. 4C). While the majority of the analysed integrin β subunits displayed a mean of R ≥ 2, talin recruitment in the case of CEA3-ITGB4, CEA3-ITGB5, and CEA3-ITGB8 was not significantly different from the negative control (Fig. 4D). Interestingly, the cytoplasmic domains of integrin β1, β2 and β3 were identical in their ability to recruit talin, with roughly 85% of all cells being positive for talin recruitment (Fig. 4E). The lack of talin recruitment in the case of CEA3-ITGB4 and CEA3–ITGB8 can be explained by their lack of the conserved NPXY/F motif (Fig. 4D,E). Surprisingly, the cytoplasmic domain of integrin β5 seemed also to be less efficient in its ability to recruit talin. Positive talin recruitment was only observed in around 62% of cells transfected with CEA3-ITGB5 and the mean R of CEA3-ITGB5 was only 2.1 (Fig. 4D,E). Taken together our results indicate that association with talin is a conserved feature of outside-in activation for most integrins. Though the possession of a NPXY/F motif is a prerequisite for talin binding, there appear to be quantitative differences in the ability of particular integrins, such as integrin β5, to efficiently recruit talin in a cellular environment.

### OPTIC reveals differences between integrin β1 and β3 in recruiting cytoplasmic proteins

**Figure 5:**
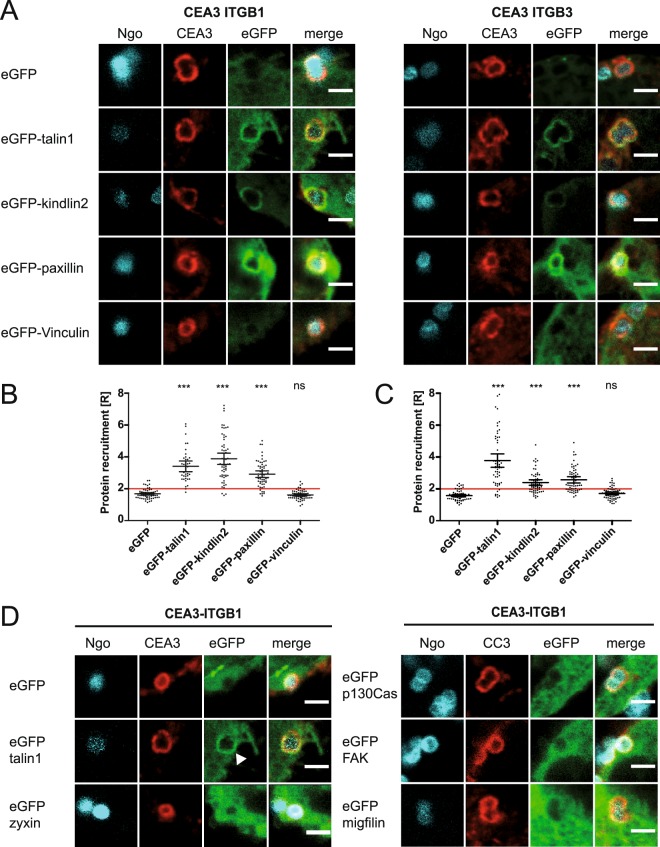
OPTIC reveals differences between integrin β1 and β3 in recruiting cytoplasmic proteins. (**A**) 293 T cells were transiently co-transfected with CEA3-ITGB1 or CEA3-ITGB3 in combination with different focal adhesion proteins tagged with eGFP. Transfected cells were seeded on poly-L-lysine coated coverslips. Cells were infected for 1 h with Pacific Blue-labeled, Opa expressing Ngo, fixed, stained for CEACAM3, and recruitment of the different proteins to CEACAM3-ITGB1 or CEA3-ITGB3 was observed. Depicted are representative micrographs of the infection sites. Bars correspond to 2 µm. (**B**,**C**) Quantification of protein recruitment to cells expressing CEACAM3-ITGB1 (**B**) or expressing CEACAM3-ITGB3 (**C**). Each data point reflects the recruitment ratio R in a CEACAM3-expressing cell with associated bacteria. Horizontal lines indicate mean values and 95% confidence intervals (whiskers) of n = 60 cells from three independent experiments. Statistically significant differences from the CEA3-ΔCT control were evaluated using one-way ANOVA, followed by Bonferroni post-hoc test (***p < 0.001, ns = not significant). (**D**) 293 T cells were transiently transfected with CEA3-ITGB1 together with the indicated, GFP-tagged focal adhesion proteins. Cells were seeded on poly-L-lysine and infected for 1 h with Pacific blue-labeled, Opa expressing *N*. *gonorrhoeae* (Ngo). After fixation, samples were stained for CEACAM3. Depicted are representative micrographs of the infection sites. Only talin shows a clear recruitment to CEA3-ITGB1 (white arrowhead), while zyxin, p130^CAS^, FAK, and migfilin are not enriched around bacterial attachment sites. Bars correspond to 2 µm.

Since our microscopic approach was able to detect differences in the extent of talin recruitment to distinct integrin cytoplasmic domains, we wondered if this analysis could be extended to additional interaction partners of the integrin β cytoplasmic tail. In particular, integrin β1 and integrin β3 share a large set of cytosolic binding partners, yet their engagement by extracellular matrix proteins has been connected to different cellular outcomes such as modulation of adhesion strength in the case of integrin β1 or adhesion maturation in the case of integrin β3^18,19^. Therefore, we compared these two integrin subunits in their ability to recruit a set of FA proteins known to be involved in integrin activation and FA maturation. As observed before, talin recruitment was comparable between CEA3-ITGB1 and CEA3-ITGB3 (R = 3.41 and R = 3.78, respectively) (Fig. 5A–C). In contrast, kindlin recruitment to the integrin β1 cytoplasmic tail was 1.6 fold higher than to the integrin β3 cytoplasmic tail (R = 3.9 and R = 2.4, respectively) (Fig. 5A–C). Interestingly, we were also able to detect paxillin recruitment to both integrin chimeras with only marginal differences (R = 2.92 for ITGB1 and R = 2.57 for ITGB3), while vinculin, a marker for mature FAs, was not recruited, neither to CEA3-ITGB1 nor to CEA3-ITGB3 (Fig. 5A–C). Further FA proteins, such as zyxin, focal adhesion kinase (FAK), p130^CAS^, and migfilin were also not recruited to the clustered integrin cytoplasmic domains (Fig. 5D). In summary, of all the proteins tested, those found in nascent adhesions were recruited to the chimeric constructs upon receptor clustering, suggesting that our assay mimics early, force-independent nascent adhesions.

### Talin recruitment is highly conserved throughout evolution

**Figure 6:**
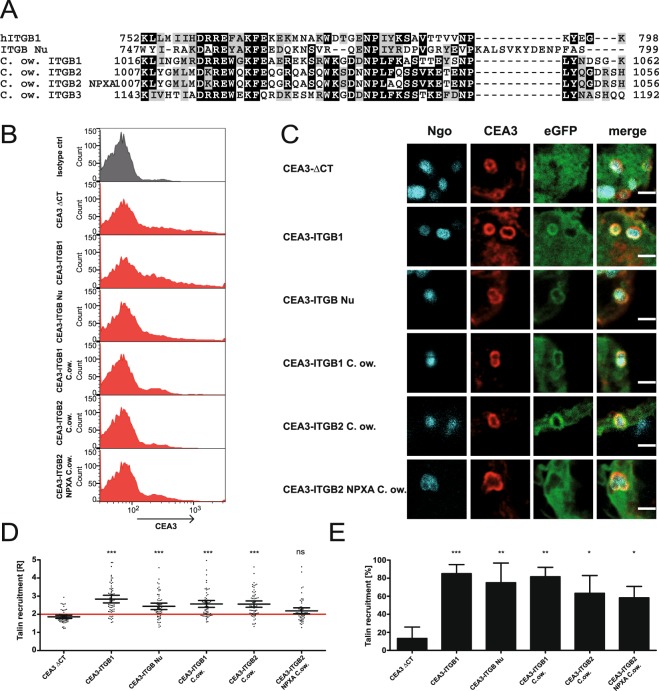
Talin recruitment is conserved throughout evolution. (**A**) Amino acid sequence alignment of evolutionary distant integrin β cytoplasmic tails in comparison to human integrin β1. Identical residues are shaded in black, highly similar residues are shaded gray. (**B**) 293 T cells were transiently co-transfected with the indicated CEACAM3-integrin fusion constructs and GFP-talin1. Cells were stained for CEACAM3 and analysed by flow cytometry. (**C**) Cells as in (**B**) were infected for 1 h with Pacific Blue-labeled Ngo, fixed, stained for CEACAM3, and talin recruitment to different CEACAM3-integrin fusion proteins was observed. Depicted are representative micrographs of the infection sites. Bars correspond to 2 µm. (**D**) Quantification of talin recruitment in cells expressing the indicated CEA3-integrin fusion proteins. Each data point reflects the talin recruitment ratio R in a CEACAM3-expressing cell with CEACAM3-bound bacteria. Horizontal lines indicate mean values and 95% confidence intervals (whiskers) of n = 60 cells from three independent experiments. Statistically significant differences from the CEA3-ΔCT control were evaluated using one-way ANOVA, followed by Bonferroni post-hoc test (***p < 0.001, ns = not significant). (**E**) Percentage of CEACAM3-expressing cells, which show a ratio of talin recruitment R ≥ 2. Statistical significance was calculated using one-way ANOVA, followed by Bonferroni post-hoc test (*p < 0.05; **p < 0.01; ***p < 0.001, ns = not significant).

Integrins were thought to be solely expressed by metazoans until genome sequencing of unicellular protists, such as the filasterean *Capsaspora owczarzaki* (C.ow) and the apusozoan protist *Amastigomonas sp*. revealed the presence of genes encoding homologues of the integrin adhesion and signalling machinery^20^. Prompted by the ancient origin of integrins and associated proteins, we wondered whether integrin regulation by talin has emerged before metazoans. Sequence alignment of integrin β subunits encoded by *C*. *owczarzaki* with human integrin β1 and the integrin β-nu subunit from *Drosophila melanogaster* revealed a high conservation of the membrane distal as well as the membrane proximal NPXY/F motifs (Fig. 6A). We used codon optimized sequences of the cytoplasmic domains from two integrin homologues found in *C*. *owczarzaki* (UniProt identifiers D7PE18-1 and D7PE19-1 designated ITGB1 C.ow and ITGB2 C.ow, respectively) and produced CEA3-ITGB C.ow fusion constructs. All C.ow integrin chimeras were expressed on the cell surface (Fig. 6B) and were able to recruit human talin with a ratio of R > 2 (Fig. 6C–E). These findings imply a functional conservation of the integrin-talin interaction. To further strengthen a presumable ancient origin of the integrin-talin interaction, we introduced a point mutation into the NPXF motif of the C.ow ITGB2 sequence. When this NPXA-mutant was tested in OPTIC assays, there was impaired recruitment of talin demonstrating that the interaction of the *Capsaspora* integrin with the cytoplasmic interaction partner is based on the same mechanistic principle. This finding also implies that the *Capsaspora* integrins might be regulated by outside-in and inside-out processes involving talin.

## Discussion

Interpretation of focal adhesion composition and dynamics in a cellular context is complicated by the fact that multiple integrins as well as diverse extracellular matrix ligands are present for any given cell type^21,22^. Indeed, focal adhesions are not homogenous clusters of one particular integrin heterodimer, but usually are composed of a combination of integrins, e.g. integrin α5β1 and integrin αVβ3 in cells adhering to fibronectin^23^. The cellular response upon integrin activation and clustering can further be influenced by crosstalk with other surface receptors, such as growth factor receptors, other cell adhesion molecules, or the glycocalyx^23,24^. These complications can be circumvented by employing chimeric integrins, which have been used before to dissect the role of the integrin cytoplasmic domain in receptor localization in non-adherent cells^25^. Inspired by this prior work, we present here a new approach to investigate the first steps in the assembly of integrin-associated focal adhesions. By using a recombinant system that is independent of natural integrin ligands and that provides temporal and spatial control over integrin clustering we can directly compare different integrin β subunits for their ability to recruit FA proteins. As the extracellular fusion partner for integrin cytoplasmic domains we chose CEACAM3, an immunoglobulin-related receptor exclusively expressed by human granulocytes. Currently, there is no endogenous ligand for this receptor known, making it unlikely that CEACAM3-containing chimeric receptors are engaged by other cellular factors or soluble serum components. Nevertheless, several high affinity ligands for CEACAM3 are known to be present on the surface of particular gram-negative bacteria, such as the gonococcus, *Neisseria gonorrhoeae*^26,27^. Therefore, CEACAM3 clustering is selectively stimulated by addition of these multivalent microbes, which in turn trigger recruitment of cytoplasmic integrin binding proteins. As the micrometer-sized bacteria can be easily identified by microscopy, even early integrin clusters can be observed and the potential enrichment of fluorescently labelled cytosolic interaction partners can be visually addressed on the single cell level.

Talin binding to integrin β1, β2, β3, β5 and β7 and the importance of the membrane proximal NPXY/F motif as the core talin binding site has been characterized before using synthetic peptides and purified recombinant proteins^28,29^. In their biochemical studies, Calderwood *et al*. added a coiled-coil sequence to the amino-terminal end of integrin cytoplasmic tails to promote dimerization. By fusing the integrin β cytoplasmic tails to the transmembrane domain of CEACAM3 and clustering with multivalent ligands, we can now show that the OPTIC approach is well suited to translate and confirm these findings in a cellular context. Interestingly, we observed weaker recruitment of talin to integrin β5, a feature that has not been seen in pulldown assays with recombinant proteins^28^. Besides its function in cell adhesion, integrin β5 is also involved in clathrin-mediated endocytosis, where the clathrin-adaptin complex is engaging the membrane proximal NPXY motif^29^. This might explain why we observe a reduced recruitment of talin in a cellular environment, where several factors might compete for binding to integrin β5. Furthermore, we could detect recruitment of talin to the cytoplasmic tail of integrin β6, which is, to our knowledge, the first experimental proof of this interaction in a human cell-based system. In the future, the incorporation of integrin α subunit cytoplasmic domains in these types of assays might provide additional insight into the behaviour and interaction partners of the complete integrin heterodimer during nascent adhesion formation and integrin activation.

Interestingly, only talin, kindlin and paxillin were recruited to the integrin β1 cytoplasmic domain upon clustering. Other FA proteins characteristic for mature FAs like vinculin, zyxin, migfilin, FAK or p130^CAS^ were not enriched around chimeric integrins. The adhesion recruitment of these proteins was previously shown to depend on the motor activity of myosin II^30^. This is in line with the hierarchical assembly model of focal adhesions^31^ and indicates that OPTIC-initiated integrin clusters mimic force-independent nascent adhesions. Clearly, gonococci as mobile ligands should not be able to impart pulling forces towards the engaged receptors. Therefore, mechanical unfolding of talin and exposure of cryptic vinculin binding sites in the carboxy-terminal domain of talin would not occur^32–34^. This could explain, why recruitment of vinculin to the clustered integrin tails is not observed during OPTIC. A further indication that bacteria-triggered clustering of integrin cytoplasmic domains does not result in extracellular-intracellular force coupling is seen by the lack of recruitment of LIM domain-containing adaptor proteins. Indeed, LIM domain proteins have been suggested to act as tension sensors and to enrich at focal adhesions in a myosinII-dependent manner^35^. In our case, LIM domain containing proteins such as migfilin and zyxin did not colocalize with the clustered chimeric receptors upon bacterial infection, further supporting the idea that OPTIC mimicks the initial, force-independent events after integrin activation. A different behaviour is observed for the LIM domain protein paxillin, which is known to associate with nascent focal adhesions in a myosinII-independent manner^30^. Paxillin strongly co-localized with the cytoplasmic domain of integrin β1 upon receptor clustering, which could be explained by the reported direct interaction of paxillin and kindlin-2^36,37^. However, as paxillin was also strongly recruited to integrin β3, where kindlin association was reduced, there might be additional routes how paxillin interacts with the integrin β3 carboxy terminus. Nevertheless, it would be highly interesting to analyse the recruitment of additional focal adhesion-enriched LIM domain proteins related to zyxin and paxillin (such as leupaxin, Hic-5, Ajuba, TRIP6, or LPP), under different force regimes (e.g. using optical tweezers) to further shed light on focal adhesion maturation.

Integrin-mediated processes like cell adhesion and cell signalling are indispensable for multicellular life and were thought to be metazoan specific. This misconception was abandoned when genome information about unicellular relatives of metazoans became available^20,38^. Interestingly, the talin and kindlin binding sites in the cytoplasmic tail of these ancient pre-metazoan integrins are highly conserved. The fact that *Capsaspora* integrin sequences were able to recruit human talin strongly suggests a functional conservation of talin-mediated activity regulation of integrins. It remains to be elucidated, if *Capsaspora* can regulate its integrin activity through both outside-in as well as inside-out activation as seen in mammals. Recently, integrin homologues were also discovered in the ichthyosporean *Sphaeroforma arctica* and the apusozoan *Amastigomonas spec*.^39^ suggesting that non-metazoan integrins can be found in various protists. Though the functional role of these integrin-related proteins in unicellular eukaryotes is currently completely unclear, we believe that our novel assay can provide valuable insight into the origin of integrin signaling.

Taken together, our results indicate that CEACAM-integrin chimeras are well suited to study integrin-associated protein complexes in a cellular context. Thereby, OPTIC can corroborate data obtained with recombinant proteins in *in vitro* assays, as the quantitative evaluation is sensitive enough to study the effect of single amino acid changes on protein complex formation. Furthermore, this experimental approach might be particularly useful to unravel the function and signaling connections of ancient integrins.

## Material and Methods

### Plasmids

Constructs encoding pEGFP-FAK, pEGFP-vinculin, and pEGFP-zyxin were as described^40^. In addition, cDNAs of human kindlin-2 (Bioscience, Nottingham, UK), paxillin and human talin1 were cloned into pDNR Dual (Clontech, Mountain View, USA). Cre/LoxP recombination was used to move sequences from pDNR Dual into GFP- or mCherry-encoding acceptor vectors for eukaryotic expression. cDNA of human integrin β1 (a gift from Michael Davidson, Addgene plasmid # 54129), human integrin β4 (a gift from Filippo Giancotti, Addgene plasmid # 16039), and human integrin β5 (a gift from Raymond Birge, Addgene plasmid # 14996) were obtained from Addgene (Watertown, MA). Human integrin β2 and human integrin β3 were acquired from RZPD (Berlin, Germany). CEACAM3-integrin β fusion constructs are summarized in Supplementary Fig. S1 and were created via PCR amplification using above mentioned templates and the following primers: integrin β1[E762-K798] BamHI sense (5′-GCGGCTATGGATCCGAATTCGCTAAATTTGAGGAAGAACGCGCCAGAG-3′), integrin β1[E762-K798] XhoI anti (5′-TATCTCGAGTTAAGTGCCCCGGTACGTG-3′), integrin β2[E734-S769] BamHI sense (5′-GCGGCTATGGATCCGAGTACAGGCGCTTTGAGAAGG-3′), integrin β2[E734-S769] XhoI anti (5′-TATCTCGAGTTAACTCTCAGCAAACTTGGGGTTCATGAC-3′), integrin β3[E752-T788] BamHI sense (5′-GCGGCTATGGATCCGAATTCGCTAAATTTGAGGAAGAACGCGCCAGAG-3′), integrin β3[E752-T788] XhoI anti (5′-TATCTCGAGTTAAGTGCCCCGGTACGTG-3′), integrin β4 [744–1822] BamHI sense (5′-GCGGCTATGGATCCGCACTTCTCCCGTGCTGCAACC-3′) integrin β4 [744–1822] XhoI anti (5′-TATCTCGAGTCAAGTTTGGAAGAACTGTTGGTCCATGTG-3′), integrin β5[E753-799] BamHI sense (5′-GCGGCTATGGATCCGAGTTTGCAAAGTTTCAGAGCGAGCGATCCAG-3′), integrin β5[E753–799] XhoI anti (5′-TATCTCGAGTCAGTCCACAGTGCCATTGTAGG-3′). PCR products were then cloned into pcDNA3.1 CEACAM3 ΔCT-HA^41^ using BamHI/XhoI restriction sites. The cDNA for cytoplasmic regions of human integrin β6, β8 and drosophila integrin βNu (NCBI accession numbers NM_000888.4, NM_002214, and NM_078884.3, respectively) as well as codon optimized *C*. *owczarzaki* beta integrins were synthesized (Eurofins, Ebersberg, Germany) and cloned into pcDNA3.1 CEACAM3 ΔCT-HA using BamHI/XhoI restriction sites. The expression constructs encoding the human CEA3-ITGB chimeras have been deposited at Addgene (plasmid IDs 124563–124571). For sequence alignments, the integrin cytoplasmic domain amino acid sequences were compiled using T-coffee (https://www.ebi.ac.uk/Tools/msa/tcoffee/) and coloured using the BoxShade tool at Expasy (https://embnet.vital-it.ch/software/BOX_form.html). ITGB4 was excluded from analysis due to the excessive length of the cytoplasmic tail.

### Antibodies

The following primary and secondary antibodies were used at indicated concentrations: mouse monoclonal anti CEACAM1, 3, 4, 5, 6 (D14HD11 from Aldevron, Freiburg, Germany) WB: 1:6000, IF: 1:200; mouse monoclonal anti-CEACAM3/CEACAM5 (COL-1 from Invitrogen) FC: 1:200; mouse monoclonal anti GFP (JL8 from Clontech, Mountain View, USA) WB: 1:6000; mouse monoclonal anti tubulin (clone E7, purified from hybridoma cell supernatants, Developmental Studies Hybridoma Bank, University of Iowa, USA) WB: 1:1000. Secondary antibodies used: horseradish peroxidase (HRP)-conjugated goat anti-mouse WB: 1:10.000; Cy5-conjugated goat anti-mouse IF: 1:200, Biotin-conjugated goat anti-mouse FC: 1:200 and AlexaFluor488-streptavidin FC: 1:200 (all from Jackson ImmunoResearch Inc., Baltimore, USA).

### Cell culture and transfection

Human embryonic kidney 293 T cells (293 T cells) were cultured in regular DMEM growth medium (Dulbecco’s Modified Eagle’s Medium, Biochrome, Merck Millipore, Berlin, Germany) supplemented with 10% calf serum (CS, Biochrome) at 37 °C and 5% CO_2_. The day before transfection, cells were seeded at 25% confluence in new 10 cm culture dishes. Cells were transfected using calcium phosphate transfection with a total amount of 5 µg plasmid DNA/dish.

### Flow cytometry

48 h after transfection, 293 T cells were trypsinized and suspended in growth medium. Samples were centrifuged at 100 g for 3 min and the resulting pellet was re-suspended in FACS buffer (PBS with 5% CS, 2 mM EDTA). 1 × 10^6^ cells per sample were incubated with a mouse monoclonal anti-CEACAM antibody for 1 h at 4 °C. After washing, cells were incubated for 30 min with a biotinylated goat anti-mouse antibody and another 30 min with AlexaFluor488-conjugated streptavidin. Cells were analysed by flow cytometry (BD LSRII, FACSDiva™ software, BD Biosciences, Heidelberg, Germany).

### Bacterial culture and staining

Non-piliated *Neisseria gonorrhoeae* strain MS11-B2.1 expressing the CEACAM3-binding Opa_52_ protein^14^ were grown on GC agar plates (Difco BRL, Paisley, UK) supplemented with vitamins, chloramphenicol (10 μg/ml) and erythromycin (7 μg/ml) (GC cam/erm) at 37 °C, 5% CO_2_ and subcultured daily. For infection, bacteria were collected from the plate using a cotton swab, suspended in PBS and stained with Pacific Blue (final concentration of 2 µg/ml) for 30 min under rotation at 37 °C. Bacteria were washed three times with PBS and the optical density of the suspension was used to estimate the number of the microorganisms according to a standard curve. Bacteria were suspended in DMEM and added to the cells at the indicated multiplicity of infection (MOI).

### Opa-protein triggered integrin clustering (OPTIC)

293 T cells were transfected with pcDNA3.1 CEA3-ΔCT or the indicated CEA3-ITGB fusion constructs (Supplementary Fig. S1) together with cDNA coding for the protein of interest fused to eGFP or mCherry. 48 h post-transfection, cells were seeded on coverslips coated with 10 µg/ml poly-L-lysine in suspension medium (DMEM + 0.25% BSA). After 2 h, adherent cells were infected with Pacific Blue-stained *Neisseria gonorrhoeae* (Opa_52_-expressing, non-piliated *N*. *gonorrhoeae* MS11-B2.1, kindly provided by T. Meyer, Berlin, Germany) at MOI 20 for 1 h in suspension medium. After 1 h, infection medium was aspirated and cells were fixed for 15 min with 4% paraformaldehyde in PBS at room temperature followed by 5 min permeabilization with 0.1% Triton X-100 in PBS. After washing with PBS, cells were incubated for 10 min in blocking solution (10% heat inactivated calf serum in PBS) and stained for CEACAM3. After washing, cells were again incubated for 10 min in blocking solution followed by secondary antibody staining. Coverslips were mounted on glass slides using Dako fluorescent mounting medium (Dako Inc, Carpinteria, USA).

### Evaluation of protein recruitment

Microscope pictures were analysed using LAS AF (Leica Microsystems, Mannheim, Germany). For calculating the recruitment ratio R, a region of interest was drawn around the outlines of the cell using the GFP channel (ROI1). A second region of interest was drawn around the clustered CEA3-ITG fusion protein using the CEA3-Cy5 channel (ROI2). The maximum intensity value of ROI2 measured in the GFP-channel was divided by the mean intensity value of ROI1 measured in the same channel yielding the recruitment ratio R. Accordingly, R indicates the fold difference of the maximum GFP intensity at the bacteria/bead attachment site over the average GFP fluorescence throughout the cell. Additionally, cells showing a bacterial-binding event with an R > 2 were counted and expressed as percentage of all cells (n = 60) analysed within this sample.

### Protein conjugation to microbeads

1 µm carboxylated polystyrene microbeads (Polysciences, Warrington, PA) were covalently coupled to proteins according to the manufacturer’s protocol using the carbodiimide method. To obtain single bead suspensions, beads were sonicated for 10 seconds and washed two times in 0.1 M carbonate buffer, pH 9.6, prior to activation with 2% carbodiimide dissolved in 0.1 M MES buffer (pH 5.6). Beads were washed two times in 0.1 M MES buffer and resuspended in 0.2 M borate buffer. 1 × 10^10^ beads were coupled to 300 µg of recombinant protein G in 1.2 ml 0.2 M borate buffer over night at 4 °C. Unreacted binding sites were blocked with 0.25 M ethanolamine in borate buffer. Beads were washed 3 times with PBS and stored at 4 °C. On day of experiment, 9 × 10^7^ protein G coupled beads were incubated with 1 µg of monoclonal CEACAM antibody or 1 µg of irrelevant mouse IgG for 1 h at 4 °C. Beads were washed 2 times with PBS, and blocked 30 min in PBS + 10 mg/ml BSA at room temperature. 2 × 10^6^ beads were centrifuged on to 2 × 10^5^ cells (300 g, 2 min, 25 °C) and incubated for 15 min at 37 °C before fixation.

### Fluorescent Microscopy and microscope settings

All images were taken from fixed specimens embedded in Dako fluorescent mounting medium (Dako Inc, Carpinteria, USA) on a LEICA SP5 confocal microscope equipped with a 63.0 × /1.40 NA oil HCX PL APO CS UV objective and analyzed using LAS AF Lite software. All images were acquired in xyz mode with 1024 × 1024 pixel format and 100 Hz scanning speed at 8 bit resolution. Fluorochromes used are Pacific Blue (excitation 405 nm, emission bandwidth: 436–476 nm); GFP (excitation 488 nm, emission bandwidth: 495–533 nm); mCherry (excitation 561 nm, emission bandwidth: 571–613 nm) and Cy5 (excitation 633 nm, emission bandwidth: 640–700 nm). Images were processed using ImageJ by applying the same brightness/contrast adjustments to all images within one experimental group.

### Acquisition of Western blot images

Images of western blots were acquired on the Chemidoc™ Touch Imaging System from Biorad using Chemiluminescence detection in signal accumulation mode. Acquired images were processed with photoshop by adjusting the levels of the whole image.

### Statistical analysis

All experiments were repeated three times. All statistical significances were determined using one-way ANOVA followed by Bonferroni post-hoc test. Statistical analyses were performed with Prism5 (GraphPad, La Jolla, CA, USA).

## Supplementary information

Supplementary Figures S1-S3

## Data Availability

The datasets used and/or analysed during the current study are available from the corresponding author on request.

## References

[1] King N (2004). The unicellular ancestry of animal development. Dev Cell.

[2] Hynes RO (2002). Integrins: bidirectional, allosteric signaling machines. Cell.

[3] Hauck CR, Agerer F, Muenzner P, Schmitter T (2006). Cellular adhesion molecules as targets for bacterial infection. Eur J Cell Biol.

[4] Barczyk M, Carracedo S, Gullberg D (2010). Integrins. Cell Tissue Res.

[5] Hughes PE (1996). Breaking the integrin hinge. A defined structural constraint regulates integrin signaling. J Biol Chem.

[6] Vinogradova O (2002). A structural mechanism of integrin alpha(IIb)beta(3) “inside-out” activation as regulated by its cytoplasmic face. Cell.

[7] Shattil SJ, Kim C, Ginsberg MH (2010). The final steps of integrin activation: the end game. Nature Rev Mol Cell Biol.

[8] Calderwood DA (2002). The phosphotyrosine binding-like domain of talin activates integrins. J Biol Chem.

[9] Anthis NJ (2009). The structure of an integrin/talin complex reveals the basis of inside-out signal transduction. EMBO J.

[10] Zaidel-Bar R, Itzkovitz S, Ma’ayan A, Iyengar R, Geiger B (2007). Functional atlas of the integrin adhesome. Nature Cell Biol.

[11] Horton ER (2015). Definition of a consensus integrin adhesome and its dynamics during adhesion complex assembly and disassembly. Nature Cell Biol.

[12] van der Flier A, Sonnenberg A (2001). Function and interactions of integrins. Cell Tissue Res.

[13] Kuespert K, Pils S, Hauck CR (2006). CEACAMs - their role in physiology and pathophysiology. Curr Opin Cell Biol.

[14] Buntru A (2011). Phosphatidylinositol-3’ kinase activity is critical for initiating the oxidative burst and bacterial destruction during CEACAM3-mediated phagocytosis. J Biol Chem.

[15] Buntru A, Roth A, Nyffenegger-Jann NJ, Hauck CR (2012). HemITAM signaling by CEACAM3, a human granulocyte receptor recognizing bacterial pathogens. Arch Biochem Biophys.

[16] Tadokoro S (2003). Talin binding to integrin beta tails: a final common step in integrin activation. Science.

[17] Calderwood DA (1999). The Talin head domain binds to integrin beta subunit cytoplasmic tails and regulates integrin activation. J Biol Chem.

[18] Schaufler V (2016). Selective binding and lateral clustering of alpha5beta1 and alphavbeta3 integrins: Unraveling the spatial requirements for cell spreading and focal adhesion assembly. *Cell Adhesion &*. Migration.

[19] Roca-Cusachs P, Gauthier NC, Del Rio A, Sheetz MP (2009). Clustering of alpha(5)beta(1) integrins determines adhesion strength whereas alpha(v)beta(3) and talin enable mechanotransduction. Proc Natl Acad Sci USA.

[20] Sebe-Pedros A, Roger AJ, Lang FB, King N, Ruiz-Trillo I (2010). Ancient origin of the integrin-mediated adhesion and signaling machinery. Proc Natl Acad Sci USA.

[21] Katz BZ (2000). Physical state of the extracellular matrix regulates the structure and molecular composition of cell-matrix adhesions. Mol Biol Cell.

[22] Zamir E (2000). Dynamics and segregation of cell-matrix adhesions in cultured fibroblasts. Nature Cell Biol.

[23] Klemke RL, Yebra M, Bayna EM, Cheresh DA (1994). Receptor tyrosine kinase signaling required for integrin alpha v beta 5-directed cell motility but not adhesion on vitronectin. J Cell Biol.

[24] Hynes RO (1999). Cell adhesion: old and new questions. Trends Cell Biol.

[25] LaFlamme SE, Akiyama SK, Yamada KM (1992). Regulation of fibronectin receptor distribution. J Cell Biol.

[26] Chen T (2001). The CGM1a (CEACAM3/CD66d)-mediated phagocytic pathway of *Neisseria gonorrhoeae* expressing opacity proteins is also the pathway to cell death. J Biol Chem.

[27] Schmitter T, Agerer F, Peterson L, Muenzner P, Hauck CR (2004). Granulocyte CEACAM3 is a phagocytic receptor of the innate immune system that mediates recognition and elimination of human-specific pathogens. J Exp Med.

[28] Calderwood DA (2003). Integrin beta cytoplasmic domain interactions with phosphotyrosine-binding domains: a structural prototype for diversity in integrin signaling. Proc Natl Acad Sci USA.

[29] De Deyne PG (1998). The vitronectin receptor associates with clathrin-coated membrane domains via the cytoplasmic domain of its beta5 subunit. J Cell Sci.

[30] Pasapera AM, Schneider IC, Rericha E, Schlaepfer DD, Waterman CM (2010). Myosin II activity regulates vinculin recruitment to focal adhesions through FAK-mediated paxillin phosphorylation. J Cell Biol.

[31] Zaidel-Bar R, Cohen M, Addadi L, Geiger B (2004). Hierarchical assembly of cell-matrix adhesion complexes. Biochem Soc Trans.

[32] Gingras AR (2005). Mapping and consensus sequence identification for multiple vinculin binding sites within the talin rod. J Biol Chem.

[33] Goult BT (2013). RIAM and vinculin binding to talin are mutually exclusive and regulate adhesion assembly and turnover. J Biol Chem.

[34] del Rio A (2009). Stretching single talin rod molecules activates vinculin binding. Science.

[35] Schiller HB, Friedel CC, Boulegue C, Fassler R (2011). Quantitative proteomics of the integrin adhesome show a myosin II-dependent recruitment of LIM domain proteins. EMBO Rep.

[36] Gao J (2017). Kindlin supports platelet integrin alphaIIbbeta3 activation by interacting with paxillin. J Cell Sci.

[37] Bottcher RT (2017). Kindlin-2 recruits paxillin and Arp2/3 to promote membrane protrusions during initial cell spreading. J Cell Biol.

[38] Suga H (2013). The *Capsaspora* genome reveals a complex unicellular prehistory of animals. Nature Commun.

[39] Sebe-Pedros A, Ruiz-Trillo I (2010). Integrin-mediated adhesion complex: Cooption of signaling systems at the dawn of Metazoa. Commun Integr Biol.

[40] Agerer F (2005). Cellular invasion by *Staphylococcus aureus* reveals a functional link between focal adhesion kinase and cortactin in integrin-mediated internalisation. J Cell Sci.

[41] Muenzner P, Rohde M, Kneitz S, Hauck CR (2005). CEACAM engagement by human pathogens enhances cell adhesion and counteracts bacteria-induced detachment of epithelial cells. J Cell Biol.

