# Supplementary material for: Clustering of integrin β cytoplasmic domains triggers nascent adhesion formation and reveals a protozoan origin of the integrin-talin interaction
Source: Sci Rep. 2019 Apr 5;9:5728. doi: 10.1038/s41598-019-42002-6 (PMC6450878; doi:10.1038/s41598-019-42002-6)
Supplement: Supplementary file 1 — Supplementary Figures S1-S3 [file 41598_2019_42002_MOESM1_ESM.pdf]

Clustering of integrin β cytoplasmic domains triggers nascent adhesion formation and reveals a protozoan origin of the integrin-talin interaction

Timo Baade[1,4], Christoph Paone[1,4], Adrian Baldrich[1], and Christof R. Hauck[1,4*]

[1]Lehrstuhl Zellbiologie, Universität Konstanz, 78457 Konstanz, Germany

[4]Konstanz Research School Chemical Biology, Universität Konstanz, 78457 Konstanz, Germany

Supplementary Figure S1

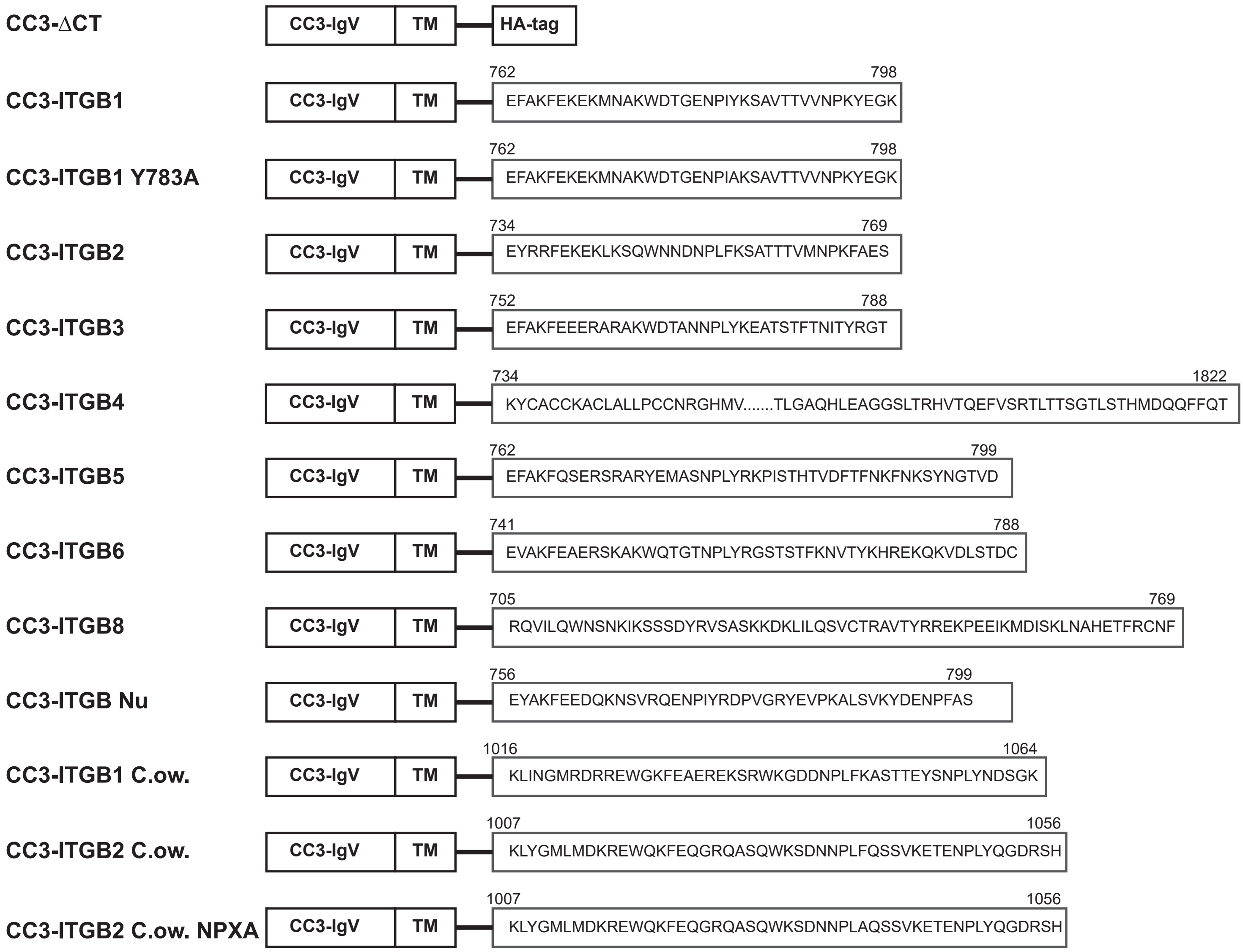

Supplementary Figure S1: Schematic overview of the CEACAM3-ITGB chimeras generated in this study.
Constructs were made in the mammalian expression vector pcDNA3.1 CEACAM3 ∆CT HA, which encodes human CEACAM3 lacking a cytoplasmic domain.
Using BamHI and XhoI restriction sites, the HA-tag encoding peptide was exchanged for different integrin beta cytoplasmic tails derived from human (ITGB1 - ITGB8), *Drosophila melanogaster* (ITGB Nu), or *Capsaspora owczarzaki* (ITGB C.ow), resulting in the indicated CEACAM3-integrin fusion proteins. Thereby, a 7 amino acid linker between the CEACAM3 transmembrane domain and the amino-terminal end of the integrin cytoplasmic domain is inserted.
Indicated amino acid positions of the integrin cytoplasmic domains correspond to the respective full-length integrins.

Supplementary Figure S2

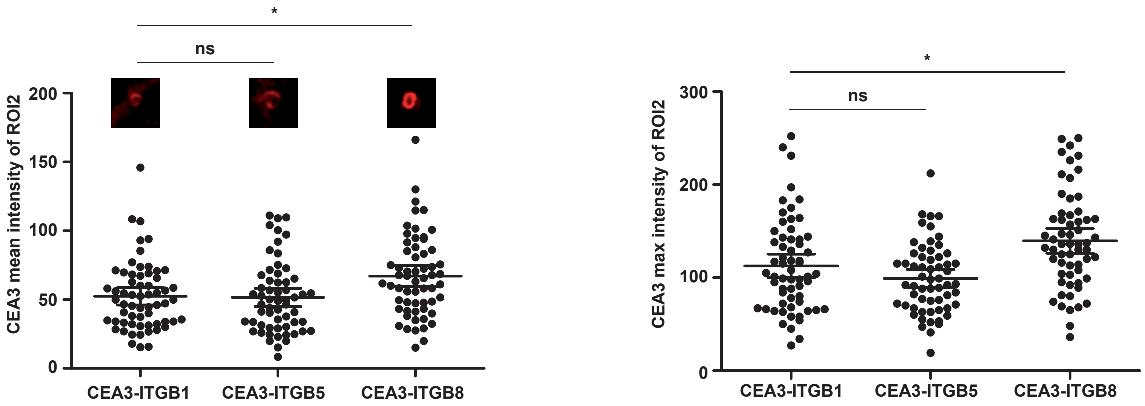

Supplementary Figure S2: Intensity analysis of clustered CEA3-ITGB fusion proteins at bacterial attachment sites

Data from Figure 4 were re-evaluated with regard to CEA3-ITGB intensity. Mean and maximum intensity of ROI2 was measured in the CEA3-ITGB Cy5 channel for three representative chimeric constructs. Data show mean values and 95% confidence intervals (whiskers) of n = 60 cells from three independent experiments.

Statistical significance was calculated using one-way ANOVA, followed by Bonferroni post-hoc test (* $p<0.01$; ns = not significant).

Supplementary Figure S3

Full scans of the Western blots shown in Figure 3B

A

Figure 3B
upper panel

The left part of this membrane contained irrelevant samples. The marker lane (M) on the left hand site, which separated samples 1-4 from the irrelevant samples on the left side, can be seen by the clear marks and are indicated by the black lines on the left. The red frame indicates the part of the blot shown in Fig. 3B.

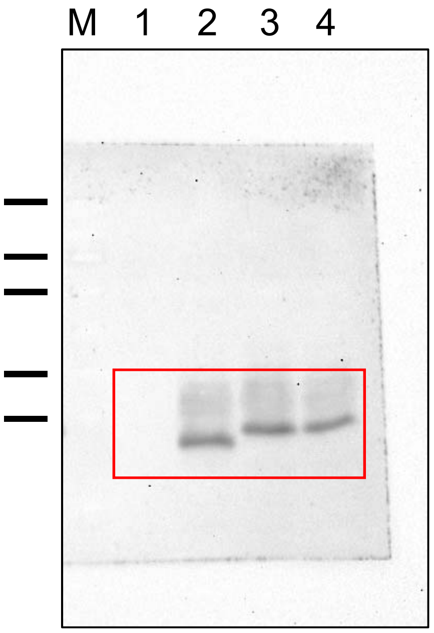

Whole cell lysate; blot α-CEACAM

B

Figure 3B
lower panel

The left part of this membrane contained irrelevant samples. The marker lane (M), which separated samples 1-4 from the irrelevant samples, is clearly visible. Samples 1-4 are derived from the same experiment as shown above and run on a separate gel. The lower part of the membrane (<40 kDa) was trimmed prior to processing with antibodies.
The red frame indicates the part of the blot shown in Fig. 3B.

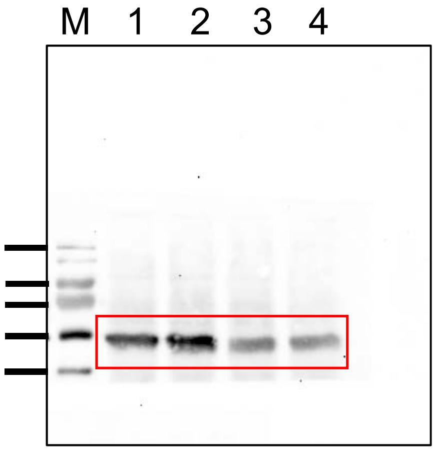

Whole cell lysate; blot α-tubulin
